# Enhancing brain-machine interface (BMI) control of a hand exoskeleton using electrooculography (EOG)

**DOI:** 10.1186/1743-0003-11-165

**Published:** 2014-12-16

**Authors:** Matthias Witkowski, Mario Cortese, Marco Cempini, Jürgen Mellinger, Nicola Vitiello, Surjo R Soekadar

**Affiliations:** Applied Neurotechnology Lab, Department of Psychiatry and Psychotherapy, University Hospital Tübingen, 72076 Tübingen, Germany; Institute of Medical Psychology and Behavioral Neurobiology, University Hospital Tübingen, 72076 Tübingen, Germany; The BioRobotics Institute, Scuola Superiore Sant’Anna, viale Rinaldo Piaggio 34,Pontedera, Pisa, Italy; Fondazione Don Carlo Gnocchi, Center of Florence, via di Scandicci 256, 50143 Firenze, Italy

**Keywords:** Brain-machine interface (BMI), Hand exoskeleton control, Electrooculography (EOG), Safety

## Abstract

**Background:**

Brain-machine interfaces (BMIs) allow direct translation of electric, magnetic or metabolic brain signals into control commands of external devices such as robots, prostheses or exoskeletons. However, non-stationarity of brain signals and susceptibility to biological or environmental artifacts impede reliable control and safety of BMIs, particularly in daily life environments. Here we introduce and tested a novel hybrid brain-neural computer interaction (BNCI) system fusing electroencephalography (EEG) and electrooculography (EOG) to enhance reliability and safety of continuous hand exoskeleton-driven grasping motions.

**Findings:**

12 healthy volunteers (8 male, mean age 28.1 ± 3.63y) used EEG (condition #1) and hybrid EEG/EOG (condition #2) signals to control a hand exoskeleton. Motor imagery-related brain activity was translated into exoskeleton-driven hand closing motions. Unintended motions could be interrupted by eye movement-related EOG signals. In order to evaluate BNCI control and safety, participants were instructed to follow a visual cue indicating either to move or not to move the hand exoskeleton in a random order. Movements exceeding 25% of a full grasping motion when the device was not supposed to be moved were defined as safety violation. While participants reached comparable control under both conditions, safety was frequently violated under condition #1 (EEG), but not under condition #2 (EEG/EOG).

**Conclusion:**

EEG/EOG biosignal fusion can substantially enhance safety of assistive BNCI systems improving their applicability in daily life environments.

**Electronic supplementary material:**

The online version of this article (doi:10.1186/1743-0003-11-165) contains supplementary material, which is available to authorized users.

## Findings

### Introduction

**Figure 1:**
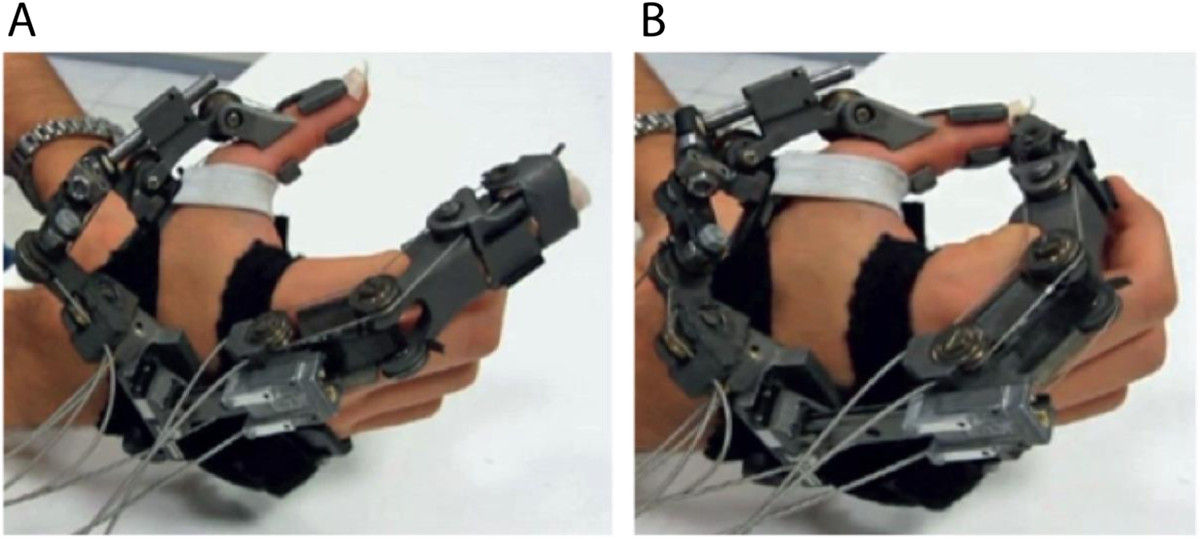
**Hand exoskeleton for grasping motions.** The illustrated device was developed by The BioRobotics Institute (Scuola Superiore Sant’Anna, Pisa, Italy) to perform opening and closing motions of a hand [2]. **A)** full opening position. **B)** full closing position.

**Figure 2:**
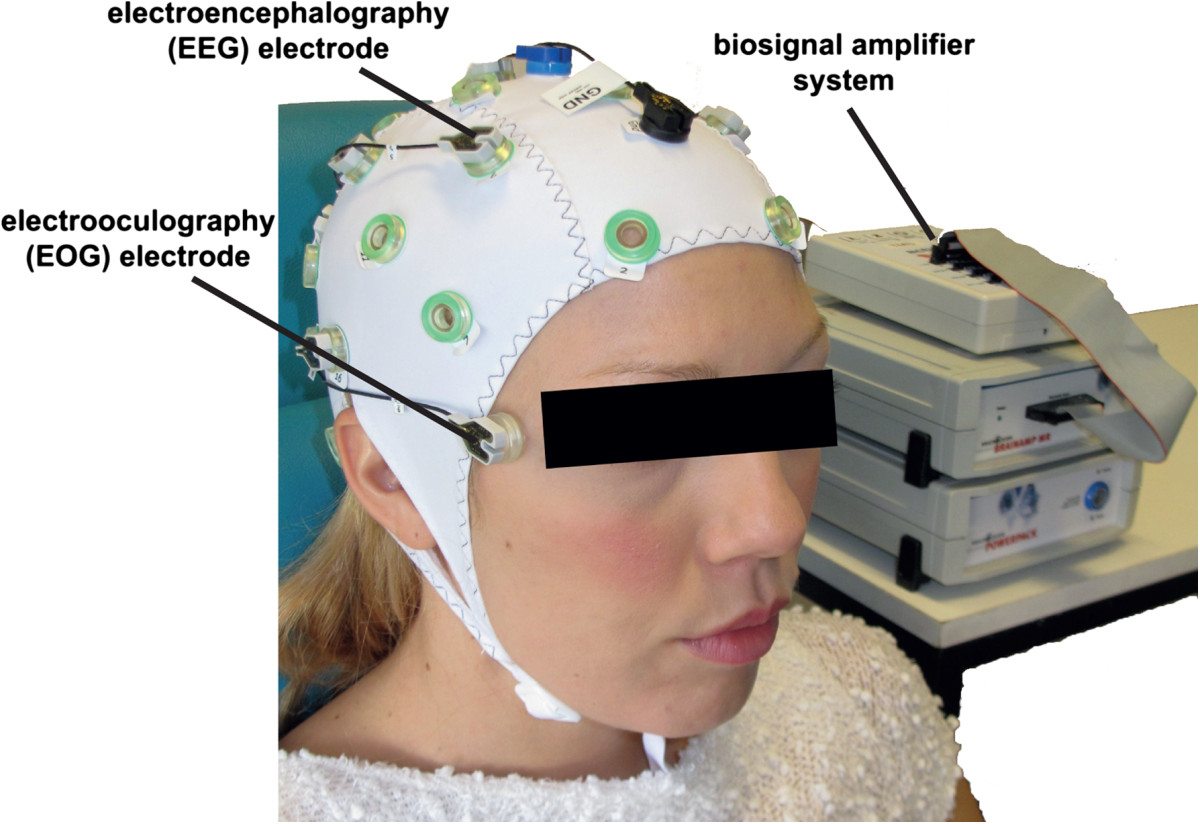
**Biosignals recorded by electroencephalography (EEG) and electrooculography (EOG) were used to control a hand-exoskeleton allowing for grasping motions.** While under condition #1 only EEG signals were used for hand exoskeleton control, both EEG and EOG signals were used during condition #2.

**Figure 3:**
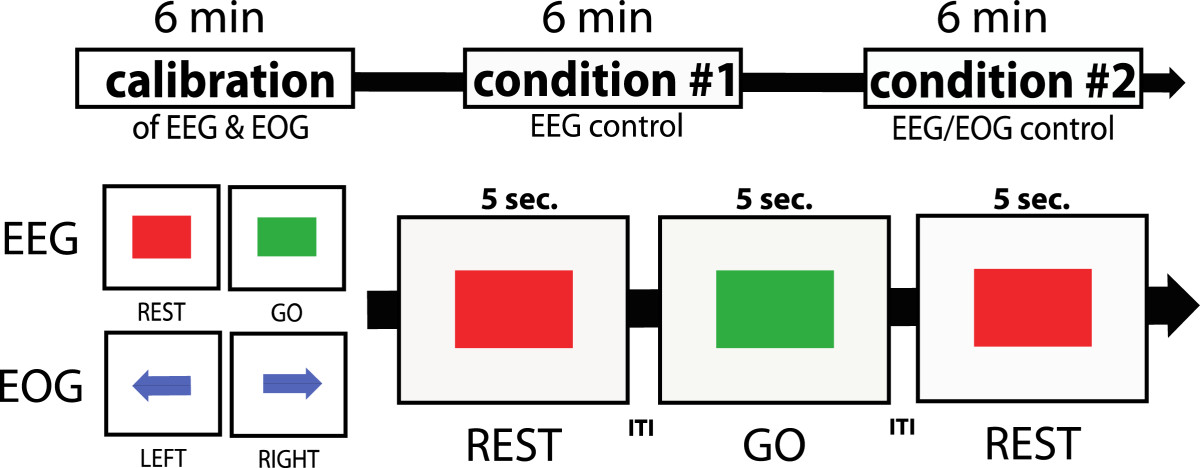
**Experimental design: after calibration, all participants controlled the BNCI system under two conditions.** During condition #1, EEG was used, while during condition #2 merged EEG and EOG signals were used for BNCI control of the hand exoskeleton. During EEG calibration, either a red square (indicating to rest) or green square (indicating to engage in motor-imagery) was shown. For EOG calibration, participants were asked to either look to the left (blue arrow to the left) or to the right (blue arrow to the right). For evaluation of BNCI control, a visual cue indicated not to move (red square) or to close the hand exoskeleton (green square) over 6 minutes in a random order. Visual indications were separated by inter-trial-intervals (ITIs) of 4-6 sec.

Real-time translation of brain activity into control signals of external devices, known today as brain-computer or brain-machine interfaces (BCI/BMI), can substantially enhance human-machine interaction (HMI) [1], e.g. allowing impaired individuals to operate assistive systems as hand prostheses or exoskeletons (Figure 1, [2, 3]). The main challenge of non-invasive BMIs relates to their accuracy in continuous detection of specific brain signals, which directly affects the system’s reliability and safety [1, 4]. Despite considerable efforts, e.g. implementation of intelligent machine learning algorithms [5, 6] or remarkable technical advances improving active BMI control [1, 3], classification accuracy of most BMI systems is still insufficient for many assistive applications, particularly those related to motor control, where misclassification can lead to unwanted actions and serious safety risks. A possible strategy to increase safety of brain-controlled assistive systems in daily life environments is to use a switch mechanism turning the BMI system off or into sleep mode when active brain control is not needed or desired [7, 8]. Moreover, recent studies combined different biosignals, e.g. EEG and EOG signals, to increase the degrees of freedom in control of external devices, e.g. to navigate a toy truck [9] or wheelchair [10]. It is unclear, though, whether fusion of bio-signals can also improve reliability and safety during ongoing, active brain control of a hand exoskeleton.

Currently, none of the available non-invasive BMI systems provide sufficient reliability for safe continuous control of motor neuro-prostheses in daily life environments.

Here we tested whether and to what extent integration of EOG can enhance such control and improve safety in controlling a hand exoskeleton for grasping motions.

### Methods

12 BNCI-naïve healthy volunteers (8 male, 4 female, mean age: 28.1 ± 3.63 years) were invited to the Institute of Medical Psychology and Behavioral Neurobiology at the University of Tübingen, Germany, to participate in a 1-hour experimental session. All participants were right handed as evaluated by the Edinburgh Handedness Inventory [11], and gave written informed consent before the session. The study protocol was approved by the University of Tübingen’s local ethics committee.

All participants were comfortably seated at a desk while EEG was recorded from 5 conventional EEG recording sites (F3, T3, C3, P3, and CZ according to the international 10/20 system) using an active electrode EEG system (Acti-cap^®^ and BrainAmp^®^, BrainProducts, Gilching, Germany) with a reference electrode placed at FCz and ground electrode at AFz. EEG was recorded at a sampling rate of 200Hz, bandpass filtered at 0.4-70Hz and pre-processed using a small Laplacian filter. EOG was recorded in accordance to the standard EOG placements at the left and right outer canthus (LOC/ROC) (Figure 2).

To rule out overt movements during motor imagery, electromyography (EMG) was recorded from the right first dorsal interosseus muscle (FDI), extensor digitorum communis (EDC), extensor carpi ulnaris (ECU) and flexor carpi radialis (FCR). Skin/electrode resistance was kept below 12 kΩ. EMG signals were sampled at 1 kHz, and passed through a high-pass filter at 2 Hz (BrainAmp ExG^®^, Brainproducts, Gilching, Germany). If EMG activity exceeded a threshold of two standard deviations above the EMG signal recorded at rest, an auditory warning tone was given and data recorded during the warning tone was excluded. A custom version of BCI2000, a multipurpose standard BMI platform [12], was used for calibration and online BNCI control. Calibration of the BNCI system was performed once at the beginning of the session and kept unvaried for the rest of the session, and comprised two parts: in the first part, participants were instructed to either rest or imagine hand grasping motions following a visual cue (red square: REST, green square: GO) displayed on a computer screen (Figure 3).

**Figure 4:**
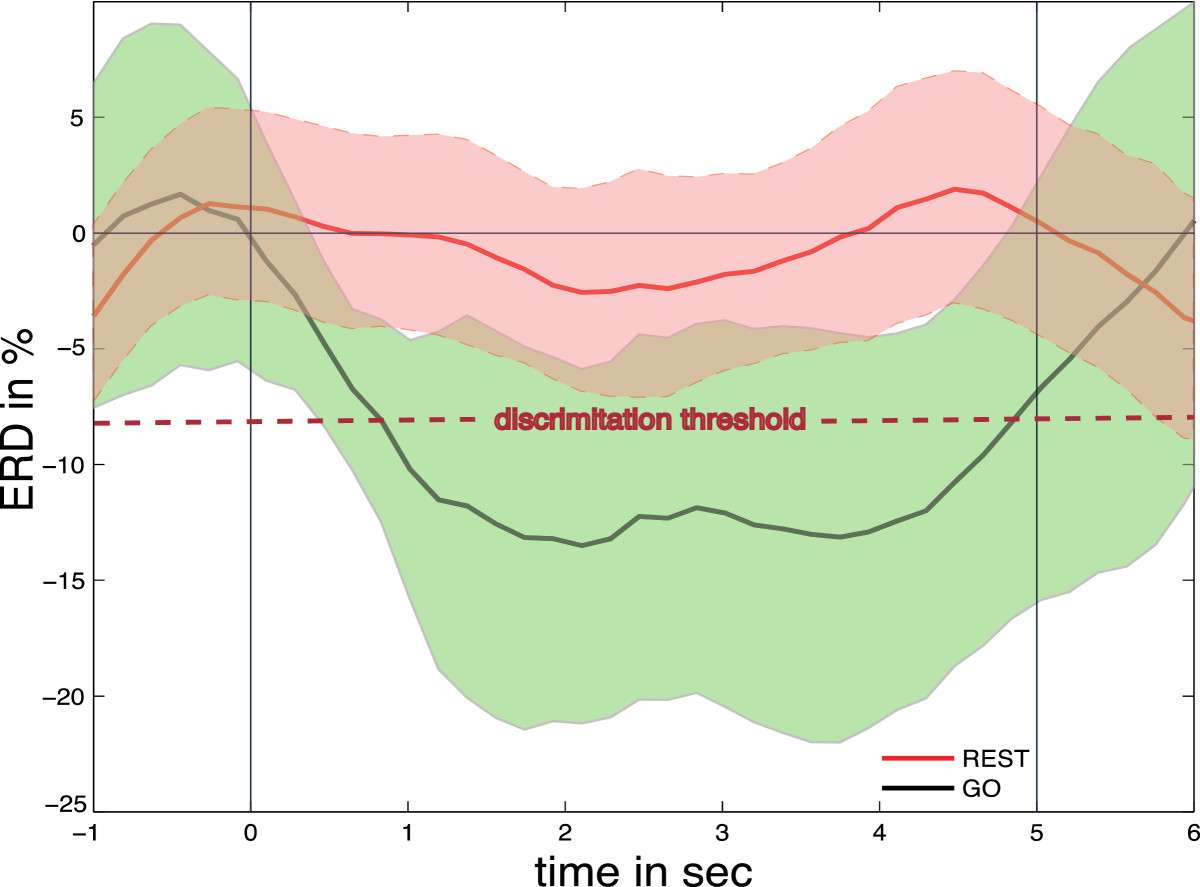
**Illustration of event-related desynchronization (ERD) of electroencephalographic (EEG) sensorimotor rhythm activity (SMR, 8-15Hz) related to motor imagery of hand closing motions in a representative participant during calibration.** The red line indicates ERD during the instruction to rest (red square presentations), while the black line indicates ERD during the instruction to imagine hand-closing motions (green square presentations). ERD was calculated relative to a reference period at -1.5 to -0.5 s before the visual cue. The 95% confidence levels are shown as red and green areas, respectively. The discrimination threshold for detection of motor imagery-related ERD for BNCI control is indicated as red dotted line.

To identify the optimal frequency for detection of motor-imagery related desynchronization of sensorimotor rhythms (SMR, 8-15Hz) of each participant, a power spectrum estimation (autoregressive model of order 16 using the Yule–Walker algorithm) was performed for each incoming sample, selecting the frequency that showed largest even-related desynchronization (ERD) during motor imagery and event-related synchronization (ERS) during rest [13, 14] recorded from C3. Based on the maximum values for ERD and ERS, a discrimination threshold was set at two-standard deviations above average SMR-ERD variance at rest, and used for later online BNCI control (Figure 4).

In the second part of the calibration, participants were instructed to move their eyes from a fixation cross in front of them to the left or to the right without turning the head upon a visual cue (arrow to the left, arrow to the right). A detection threshold for full left and right eye movements was set at two standard deviations (SD) below the average EOG signal recorded during presentation of the visual cue.

For BNCI control, participants were instructed to look at their hand affixed to a motorized hand exoskeleton and to use visuo-kinesthetic motor imagery of right-hand grasping motions to initialize exoskeleton-driven movements [14].

The reliability and safety of the BNCI system was evaluated during two conditions: during condition #1, participants used only EEG, while during condition #2, participants used EEG and EOG signals. During both conditions, a visual signal randomly indicated the user to either close (green square) or not to move the device (red square): the two indications were given 24 times each in total separated by inter-trial intervals (ITIs) of 4–6 seconds throughout a time window of approximately 6 minutes (Figure 3). Each indication was displayed for 5 seconds after which the device was driven back to open position. Re-setting the exoskeleton into open position required one second. During BNCI control, EEG signals were processed in 100 ms time windows (sample blocks). Each trial consisted of 50 sample blocks in which the BNCI software evaluated if the SMR-ERD value underwent a given threshold (ERD detection threshold) that was set at two standard deviations above average SMR variance at rest. A complete closing of the exoskeleton was only achieved if movement intention was detected during 43 sample blocks per trial leaving the participant 700 ms after presentation of a green square to initiate SMR-ERD that exceeded the ERD detection threshold. The time delay between the brain signal and the actual movement of the hand exoskeleton due to signal transmission and processing was approximately 125 ms. The speed of the exoskeleton was set to allow a full closing motion within 5 seconds.

During condition #2, exoskeleton motions could be interrupted and reset to neutral (full open) position when EOG signals exceeded the EOG detection threshold. During the trials, the participants were free to use either full left or right eye movements to reset the exoskeleton. After the session, all participants were asked to report any difficulties or discomforts during the control of the device.

To evaluate BNCI control and safety across participants and conditions, motions in % relative to a full closing during presentation of either the green or red square were calculated for each condition. A grasping motion was defined as successful if the hand exoskeleton closed the hand more than 50% while the green square was displayed. This value was chosen under the assumption that the average dimension of the most representative objects grasped in daily life range at approximately 50% of the user’s full hand span. Control of the device was defined as successful if average hand-closing motions during green square presentations exceeded more than 60% of full closing motions (i.e. a movement time > 2.6 seconds per trial) reflecting that a highly significant statistical difference (p < 0.01) of SMR-ERD between task and rest states was reached within this trial. A violation of the safety criterion was defined as a closing motion that exceeded 25% of a full hand closing while a red square was displayed. This value was chosen under the assumption that most daily life objects that are grasped are smaller than 75% of a full hand span so that hand exoskeleton-driven motion could be interrupted within one second before any force is being applied to the object. Participants were not aware of these definitions and received no feedback of performance related to these values.

To evaluate the accuracy of BNCI control under each condition, the sensitivity index (SI, equation 1) providing a measure of discriminability based on true and false positive classifications for each participant was calculated and averaged for each condition.

1 $$d^{\prime }={Ζ}_{\text{true}-\text{positive}-\text{class}.}-{Ζ}_{\text{false}-\text{positive}-\text{class}}$$

To improve comparability and as indicator of the EOG features’ impact on BNCI control and safety, also false positive classifications during ITI’s were calculated for both conditions. Statistical significance was assumed when p < 0.05.

### Results

#### Reliability

**Figure 5:**
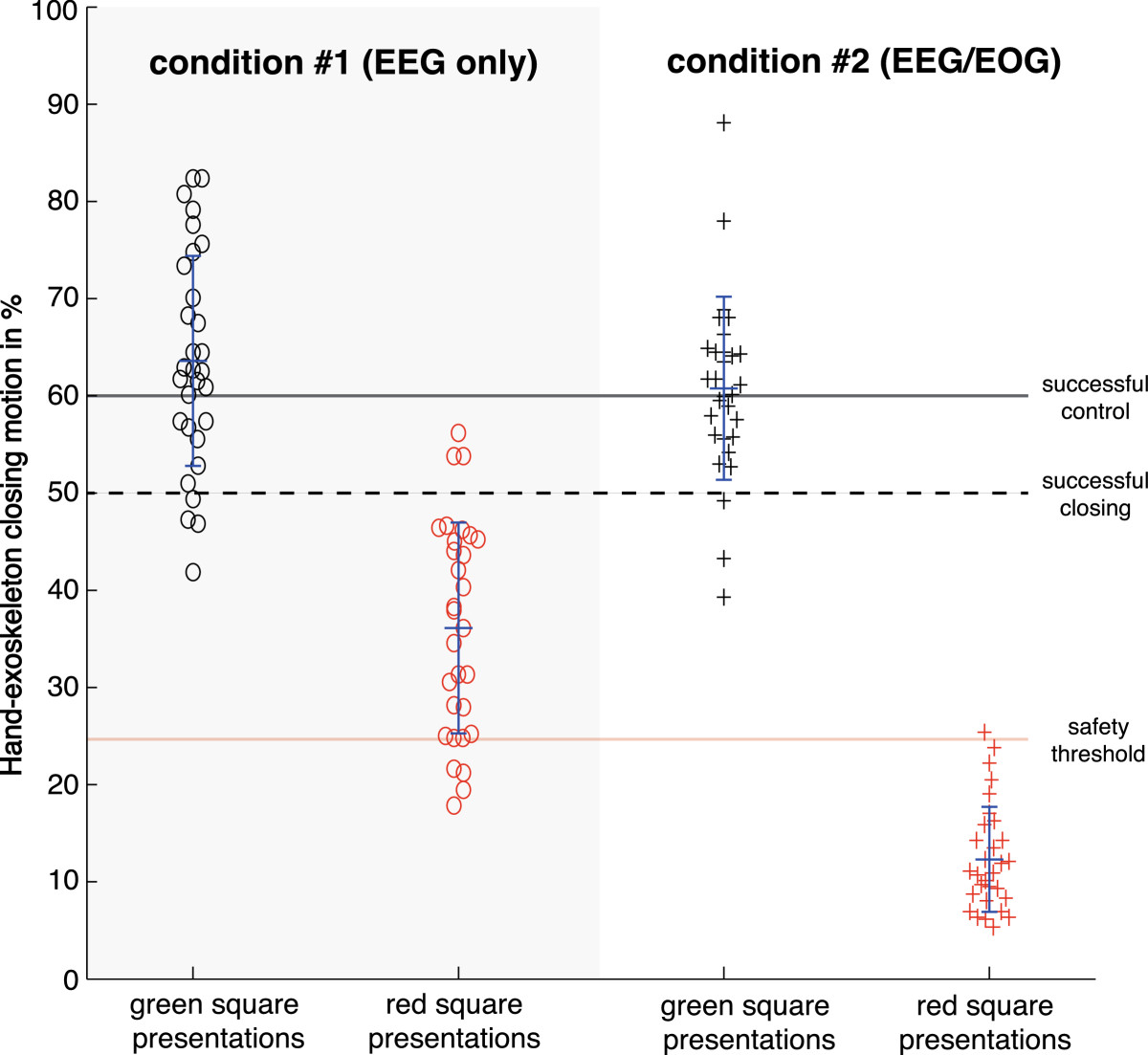
**Hand exoskeleton-closing motions in % relative to a full closing motion during EEG control (condition #1, left side) and hybrid EEG/EOG BNCI control (condition #2, right side) averaged across all participants while green or red squares were presented.** Participants were instructed to close the hand exoskeleton during green square presentations (black circles/crosses), and not to move during red square presentations (red circles/crosses). All participants were able to successfully close the device and reached successful control during green square presentations. However, during condition #1, the safety threshold (set at 25% closing motions during red square presentations) was often exceeded, but only once under condition #2.

All participants showed significant SMR-ERD during motor imagery and reached successful control of the BNCI system under both conditions. During presentation of the green square, the exoskeleton was closed in average by 63.59 ± 10.81% under condition #1 (EEG only) and 60.77 ± 9.42% under condition #2 (hybrid EEG/EOG control). While the exoskeleton closed the participants’ hand during red square presentations by 36.11 ± 10.85% under condition #1 (EEG only), the participants’ hand was closed by 12.31 ± 5.39% in average under condition #2 (hybrid EEG/EOG control) (Figure 5). During condition #2, participants used EOG signals in average in 60.9 ± 19.76% of trials.

A rmANOVA revealed a significant main effect for factors ‘motion’ (F(1,40) = 295.77, p < 0.01) and ‘condition’ (F(1,40) = 10.36, p < 0.01), and their interaction (F(1,40) = 11.99, p < 0.01). While post-hoc t-tests indicated a significant increase in motion between green and red square presentations in condition #1 (p < 0.001) and condition #2 (p < 0.001), no significant difference in motion between condition #1 and condition #2 was found when the green square was shown (p = 0.301). In contrast, when the red square was shown, motions under condition #2 were significantly smaller compared to condition #1 (p < 0.001). Calculation of the SI showed a significant difference between condition #1 (SI: 0.893 ± 0.485) and condition #2 (SI: 1.546 ± 0.258) (p < 0.001) indicating improved accuracy in BNCI control using the hybrid EEG/EOG approach. While false positive classification rates during ITI and red square presentations were not significantly different under condition 1 (p = 0.364), EOG inclusion in condition 2 resulted in a significant difference (p < 0.01) and drop of false positive classification rates reflecting improved BNCI control and safety (Table 1).

**Table 1:** **True and false positive classification rates across conditions**

	Condition 1	Condition 2
True positive rate in % (green square)	63.59 ± 10.81	60.77 ± 9.42
False positive rate in % (ITI)	28.72 ± 11.02	22.79 ± 9.43
False positive rate in % (red square)	36.11 ± 10.85	12.31 ± 5.39

#### Safety

Under condition #1 (EEG only), the safety criterion was frequently violated across all participants (in 45.91 ± 26.8% of red square presentations) and some exoskeleton motions reached up to 62% of a full closing motion. Under condition #2 (hybrid EEG/EOG control), safety violations occurred in only 10.14 ± 0.3% of red square presentations and the maximum closing motion during a red square presentation stopped at 28%.

#### Feedback from the participants

None of the participants reported any discomfort or difficulties during BNCI control. After the experiment, all participants stated that control under condition #1 was more difficult compared to condition #2, and 4 participants reported that unintended motions during condition #1 resulted in some dissatisfaction and frustration over time.

### Discussion

This is the first study that investigated whether fusion of EEG and EOG signals can enhance reliability and safety of continuous brain control of a hand exoskeleton performing grasping motions. While all participants successfully reached control under both conditions, inclusion of EOG control under condition #2 significantly reduced unintended hand exoskeleton motions. While under condition #1 unintended motions frequently exceeded 25% of a full closing motion, the violations of the safety criterion were substantially decreased when EOG control was included under condition #2. This suggests that fusing different biosignals represents a powerful strategy to increase usability and safety of non-invasive assistive motor neuro-prosthesis, for example to control an exoskeleton performing grasping motions in daily life environments. The introduced strategy’s daily life applicability could be even further improved by using a switch mechanism based, for instance, on a sequence of EOG signals (e.g. full eye movements to the left followed by eye movements to the right) turning BNCI control into sleep mode when necessary.

While the main rational for using motor imagery in the described paradigm was to provide intuitive control over a hand exoskeleton in which an imagined or attempted movement of a paralyzed hand becomes translated into a matching motion of an assistive device, use of other brain signals, e.g. SSVEPs or P300 [15] may provide better classification performance. Control of these brain signals, though, is less intuitive and depends on external stimulation that might distract from the object manipulated by the exoskeleton.

In the present study, participants had no previous experience with the use of EOG signals for BNCI control and familiarized with EOG control only during the calibration procedure at the beginning of the session. While eye movements are often left intact in patient populations with severe motor disabilities, e.g. stroke or spinal cord injuries (SCI), validity of these results and their dependence on various factors, e.g. cognitive capacity, attention span or alertness should be investigated in future studies. Also, it is conceivable that hybrid EEG/EOG BNCI control can be improved beyond the level demonstrated in this study if effective training protocols [14, 16], or advanced decoding algorithms, e.g. based on Riemannian geometry [17], discriminative models [18] or machine learning are applied.

## Authors’ original submitted files for images

Below are the links to the authors’ original submitted files for images. Authors’ original file for figure 1 Authors’ original file for figure 2 Authors’ original file for figure 3 Authors’ original file for figure 4 Authors’ original file for figure 5
