# Supplementary figures and images for: Enhancing brain-machine interface (BMI) control of a hand exoskeleton using electrooculography (EOG)
Source: J Neuroeng Rehabil. 2014 Dec 16;11:165. doi: 10.1186/1743-0003-11-165 (PMC4274709; doi:10.1186/1743-0003-11-165)

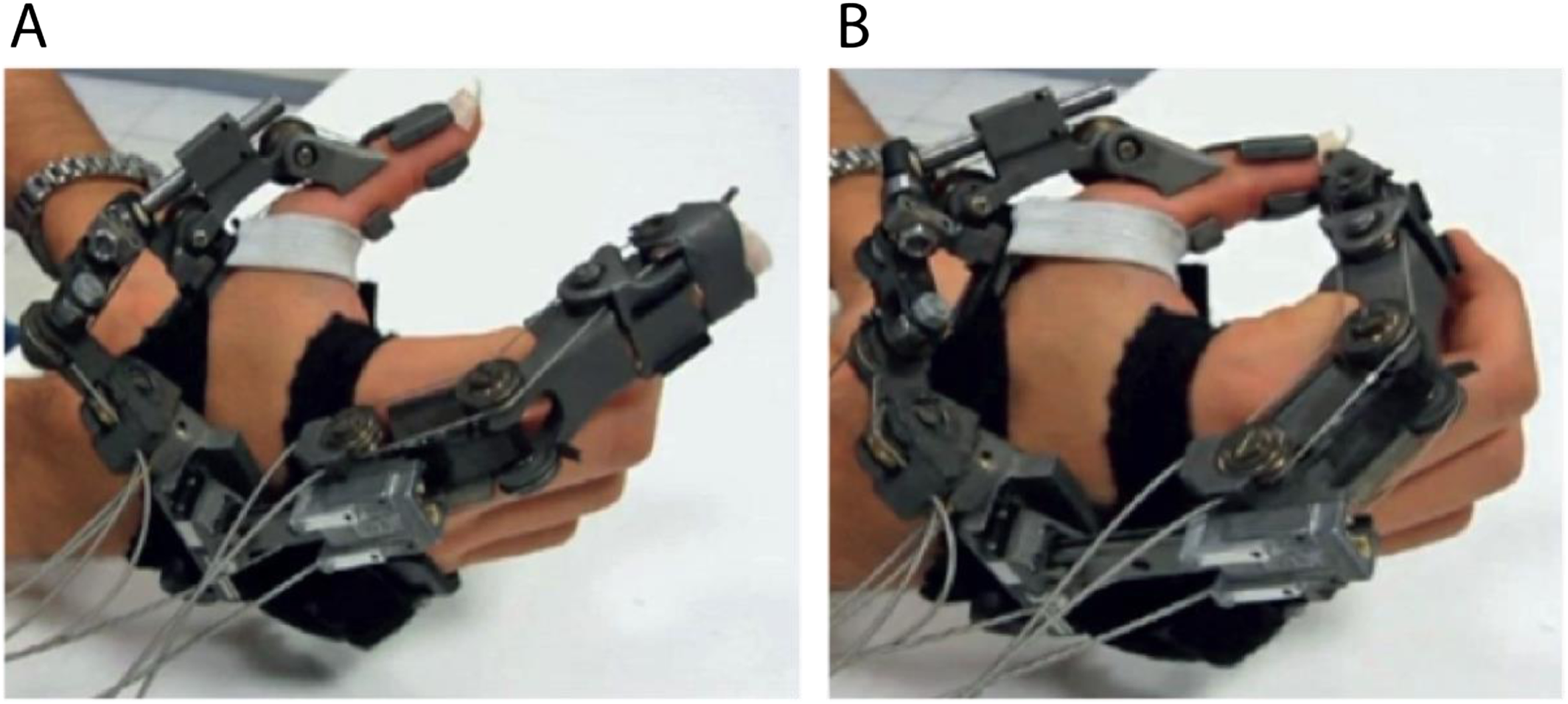

Supplement: Supplementary file 1 — Authors’ original file for figure 1 [file 12984_2014_682_MOESM1_ESM.tif]

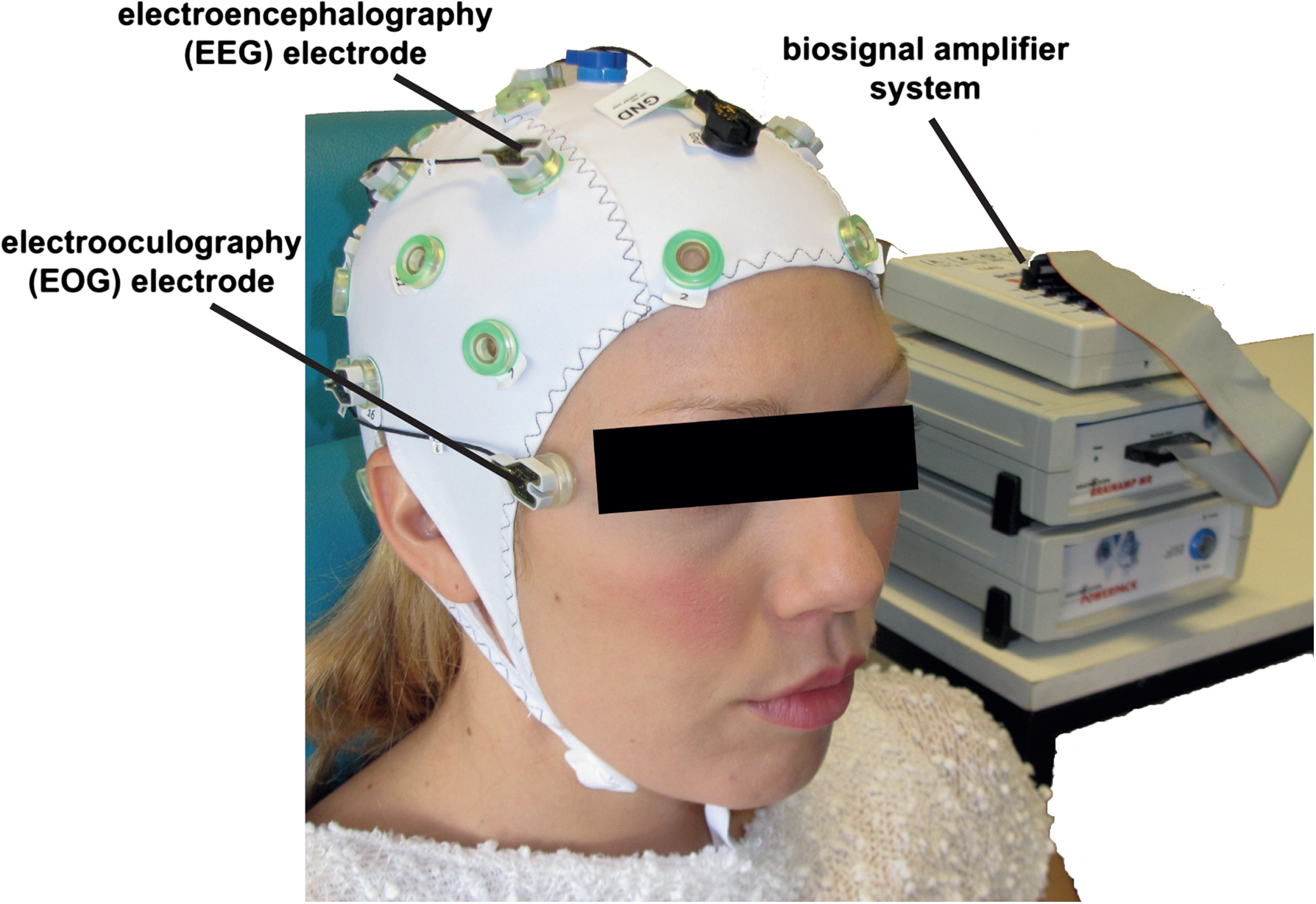

Supplement: Supplementary file 2 — Authors’ original file for figure 2 [file 12984_2014_682_MOESM2_ESM.tif]

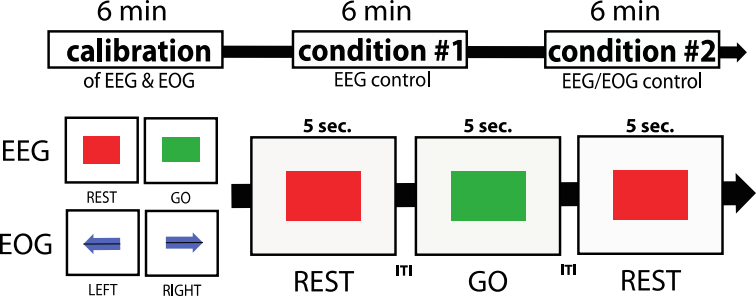

Supplement: Supplementary file 3 — Authors’ original file for figure 3 [file 12984_2014_682_MOESM3_ESM.pdf]

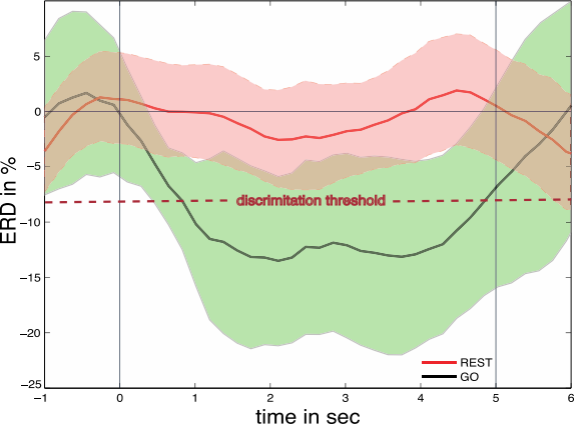

Supplement: Supplementary file 4 — Authors’ original file for figure 4 [file 12984_2014_682_MOESM4_ESM.pdf]

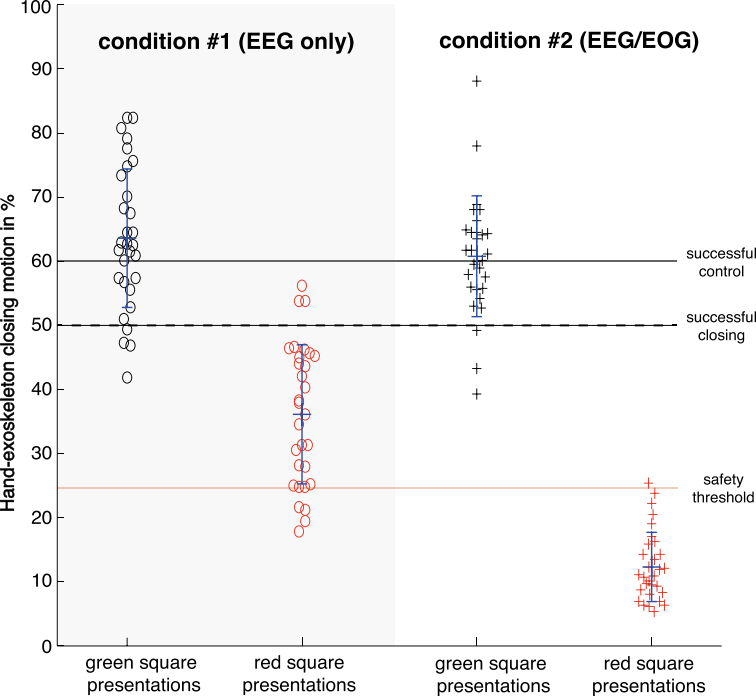

Supplement: Supplementary file 5 — Authors’ original file for figure 5 [file 12984_2014_682_MOESM5_ESM.pdf]
